# Exogenous estradiol does not regulate daily metabolic rhythms underlying diet-induced obesity in male mice

**DOI:** 10.1371/journal.pone.0343513

**Published:** 2026-03-17

**Authors:** W. Brad Osborne, Oliver Vöcking, Victoria M. Alvord, Oluwabukola B. Omotola, Yuriko Katsumata, Julie S. Pendergast

**Affiliations:** 1 Department of Biology, University of Kentucky, Lexington, Kentucky, United States of America; 2 Department of Biostatistics, University of Kentucky, Lexington, Kentucky, United States of America; Pennsylvania State University Hershey Medical Center, UNITED STATES OF AMERICA

## Abstract

Male mice fed high-fat diet become obese, but female mice are resistant to diet-induced weight gain. We previously found that circulating estradiol in females protects their daily rhythms from disruption by high-fat feeding to prevent diet-induced obesity. The goal of this study was to determine the effects of estradiol on daily metabolic rhythms in male mice. Male C57BL/6J mice were treated with estradiol and fed high-fat diet for 2 weeks. We measured the effects of high-fat diet feeding on daily rhythms of eating behavior and locomotor activity, and on the phases, or timing, of circadian rhythms in central and peripheral tissues. We found that males treated with estradiol had lower blood glucose when fed high-fat diet than males treated with vehicle even though there were no effects of estradiol treatment on body weight and adiposity. There was no effect of estradiol on the daily rhythm of eating behavior as it was low-amplitude or arrhythmic during high-fat diet feeding in both vehicle- and estradiol-treated males. Locomotor activity rhythms were also unaffected by estradiol treatment. Likewise, the phases of circadian rhythms in the suprachiasmatic nucleus (SCN), liver, muscle, and other peripheral tissues were not altered by estradiol treatment. Thus, treatment of male mice with estradiol did not protect daily rhythms from disruption by high-fat diet, as it does in females. Together these data suggest that the mechanisms underlying sex differences in daily metabolic rhythms are complex and may require both developmental and adult exposure to hormones.

## Introduction

Obesity increases the risk of cardiovascular disease, type 2 diabetes, and cancer, which are leading causes of death worldwide [1–5]. Obesity and its comorbidities differ in men and women [6–8]. Circulating estrogens in women have been implicated as the primary factor underlying sex differences in cardiometabolic diseases [9–11]. Premenopausal women are relatively protected from the negative consequences of obesity such as metabolic syndrome and heart disease [8,12,13]. After menopause, the risk of life-threatening obesity-related disorders such as cardiovascular disease and stroke increases dramatically [12,14,15]. While treating postmenopausal women with estrogens is complicated and depends on the timing of first treatment (e.g., number of years after menopause), most studies show that estradiol replacement reduces the risk of heart disease and diabetes in postmenopausal women [16–22]. It is unclear whether estrogens regulate obesity only in females, or if a similar effect occurs in males. Understanding the sex-specific effects of estrogens on metabolism is critical for developing therapeutics for metabolic disorders.

Rodent models can be used to study sex differences in diet-induced obesity. Male C57BL/6J mice fed high-fat diet become obese and develop markers of type 2 diabetes including hyperglycemia and hyperinsulinemia [23,24]. In contrast, female C57BL/6J mice are resistant to high-fat diet-induced obesity and hyperglycemia [25–27]. When females are ovariectomized to remove virtually all circulating estrogens, they become susceptible to diet-induced obesity and rapidly develop a type 2 diabetes phenotype [25,28]. This sex difference in diet-induced obesity and metabolic dysfunction in C57BL/6J mice is regulated by circulating estrogens in females. This similarly models cardiometabolic phenotypes in men and women.

Circadian rhythms are differentially affected by high-fat diet feeding and this contributes to the sex difference in obesity in mice. Circadian rhythms are 24-h cycles of behavior and physiology that are entrained, or synchronized, to environmental cycles. Male mice have robust, high amplitude eating rhythms when fed regular chow and they eat mostly during the dark phase [29–31]. High-fat diet feeding markedly decreases the amplitude of eating rhythms and males eat more evenly across the light and dark phases [29,31]. In contrast, female mice maintain high amplitude eating rhythms when fed high-fat diet [25,26]. The removal of circulating estrogens in female mice disrupts eating behavior rhythms and they become susceptible to diet-induced obesity [25,26]. Estradiol replacement in ovariectomized female mice restores high amplitude eating behavior rhythms and inhibits diet-induced obesity [25]. Thus, circulating estrogens in female mice protect daily eating rhythms from disruption by high-fat feeding and reduce obesity.

There are also sex differences in the effects of high-fat diet on circadian organization, which is the phase relationship between tissue clocks [32]. This organization is coordinated by the SCN, which receives photic information directly from the retinas and coordinates the phases, or timing, of circadian clocks located in nearly every tissue in the body [32,33]. While circadian clocks in most tissues are not altered by high-fat diet feeding, the phase of the liver clock is shifted earlier (i.e., advanced) by 5 hours in males fed high-fat diet compared to males fed low-fat diet [29]. In contrast, there is no effect of high-fat diet feeding on the phase of the liver clock in female mice [26]. The phase of the liver clock is regulated by estrogens in females since the liver phase was advanced by 4h in ovariectomized females fed high-fat diet compared to females treated with estradiol [25]. Circulating estradiol therefore regulates circadian organization in female mice, but it is not known whether estrogens can regulate the liver circadian clock in male mice.

Circulating estrogens have striking effects on the regulation of circadian rhythms underlying diet-induced obesity in female mice. However, it is not known whether estrogens can perform these same functions in male mice. It is important to study the effects of estradiol on circadian rhythms underlying obesity in males because it is critical for probing the mechanisms underlying sex differences in metabolism. Sex differences are conferred, in part, by the actions of circulating sex hormones during development (organizational effects) and/or during adulthood (activational effects). Treating male mice with estradiol in adulthood can elucidate whether its effect on a specific behavior or physiological process is solely attributed to activational effects. Thus, the objective of this study was to determine whether treatment with exogenous estradiol regulates daily rhythms underlying diet-induced obesity in male mice.

## Materials and methods

### Animals

All procedures were conducted in accordance with protocols 2015−2211 and 2021−3842 that were reviewed and approved by the University of Kentucky Institutional Animal Care and Use Committee. Heterozygous C57BL/6J PERIOD2::LUCIFERASE mice (originally obtained from Dr. Joseph Takahashi and backcrossed with C57BL/6J mice from The Jackson Laboratory for 30–36 generations) were crossed with C57BL/6J wild-type mice (stock #000664 from The Jackson Laboratory) to generate male heterozygous PER2::LUC and wild-type mice used for experiments (backcross generations N30-36). All breeders and weanlings were housed in 12L:12D and fed a standard Teklad rodent chow (2918, 18% kcal fat) and water *ad libitum*. Offspring were weaned and group-housed (2–5 male mice/cage) at 3 weeks old. Mice were genotyped by collecting tail snips and measuring luminescence with a luminometer.

### Experimental protocol

At 7 weeks old, male mice weighing 23–26 grams were randomized to be implanted subcutaneously with Silastic tubing that contained either vehicle (sesame oil, Sigma Aldrich, n = 32) or 17β-estradiol (estradiol in sesame oil, Sigma Aldrich, n = 26). PER2::LUC and wildtype mice were equally divided between groups. For Silastic tubing implantation surgeries, mice were anesthetized with isoflurane and then administered analgesic (Ketofen or Meloxicam, subcutaneous, 5–10 mg/kg) before surgery and following surgery. Silastic tubing (inner diameter: 1.98 mm; outer diameter: 3.18 mm; length of tubing: 18 mm; length of plugs: 5 mm each, Dow Corning) containing either 250 μg estradiol or sesame oil was inserted subcutaneously through a lateral incision inferior to the neck and positioned in the mid-dorsal region. Skin incisions were closed with metal wound clips, which were removed 1 week after surgery.

After surgery, mice were single-housed in cages (33 x 17 x 14 cm) in light-tight boxes in 12L:12D (white LEDs, light-intensity 250–350 lux) and fed a low-fat diet (10% kcal fat, 3.85 kcal/g, Research Diets D12450K) for 1 week. At 8 weeks old, mice were fed high-fat diet (45% kcal fat, 4.73 kcal/g, Research Diets D01060502) for 2 weeks. During the experiment, running wheels in cages were locked (could not rotate) and food and water were available *ad libitum*. Body weight and food intake were measured weekly during the 3 hours before lights off (Zeitgeber time ZT9–12 where ZT0 is light on and ZT12 is lights off). At 10 weeks old, wild-type mice were fasted for 7–8 hours starting at ZT2-ZT3 and euthanized at ZT9–10 by cervical dislocation followed by decapitation and fasting blood glucose was measured with a glucometer (Aviva Accu-check) from trunk blood. Body composition of wild-type mice was measured with EchoMRI. PER2::LUC heterozygous mice were not fasted and instead their tissues were cultured for bioluminescence analysis (described below).

### Eating behavior rhythm analysis

Infrared video cameras (HD 48Led 940nm Outdoor CMOS 800TVL IR-Cut Dome camera waterproof IF CCTV) continuously recorded mouse behavior that was stored on a security system DVR (Lorex technology MPX HD 1080p Security System DVR). Eating behavior events were analyzed in 1-min bins where 1 represented an eating event and 0 represented no eating event as previously described [29]. Circular histograms (in 10-min bins) were generated for days 5–7 (last 3 days of low-fat diet feeding) and days 19–21 (last 3 days of high-fat diet feeding) of the experiment using Oriana 4.02 software (Kovach Computing Services). For each day of eating behavior, the mean vector was determined using Rayleigh’s uniformity test. If the data were not uniformly distributed (*p* < 0.05), then a daily rhythm was present. The amplitude was defined as the length of the vector because it describes the consolidation of eating events. The phase was defined as the direction of the vector because it describes clustering around a specific time of day. If the data were uniformly distributed (*p* > 0.05), then eating behavior was arrhythmic, the amplitude (mean vector length) was 0, and there was no value for phase. Three days of eating behavior rhythms were averaged for each mouse during low-fat diet feeding or high-fat feeding.

### Locomotor activity rhythm analysis

General locomotor activity was measured continuously with passive infrared sensors (Adafruit) and collected by ClockLab Acquisition software (Actimetrics). Data were plotted as single-plotted actograms (6-min bins, inner scale: 35, outer scale: 70) with ClockLab software. Locomotor activity rhythms were analyzed on day 7 (last full day of low-fat diet feeding) and day 21 (last full day of high-fat diet feeding). Oriana software was used to make circular histograms and analyze the data with Rayleigh’s uniformity tests as described above. All mice had rhythmic locomotor activity *(*Rayleigh tests *p* < 0.05). The lengths and directions of the vectors were the amplitudes and phases, respectively, of the activity rhythms as described above.

### Bioluminescence rhythms in ex vivo tissues

At 10 weeks old, heterozygous PER2::LUC mice were euthanized within 1.5 hours of lights out (ZT 10.5–12) by cervical dislocation without anesthesia and the aorta, kidney, liver, lung, soleus muscle, pituitary, suprachiasmatic nucleus (SCN), spleen, and white adipose tissue (WAT) were cultured as previously described [29,33,34]. Bioluminescence was measured from tissues using the LumiCycle apparatus (Actimetrics) for 1 minute every 10 minutes. Rhythms of bioluminescence were detrended and smoothed (30-min adjacent averaging) using LumiCycle Analysis software. ClockLab Analysis software was used to measure phases (acrophases) of bioluminescence rhythms in the interval between 12 and 36 hours in culture. The periods of bioluminesecence rhythms were measured from a line fit to the acrophases of the first 3 cycles in culture (ClockLab Analysis).

### Statistical analyses

Data are presented as mean ± SEM. Significance was ascribed at *p* < 0.05. A two-way repeated measures ANOVA with Bonferroni correction was used to analyze the effect of estradiol treatment on body weight. Student’s two-tailed *t*-*t*ests (when data were normally distributed and had equal variance) or Mann-Whitney tests (when data were not normally distributed or had unequal variance) were used to compare adiposity, fasting blood glucose, cumulative food intake, total activity, and the phases and periods of PER2::LUC rhythms in each tissue between males treated with oil and those treated with estradiol. Two-way ANOVAs (diet*estradiol treatment) were used to determine whether amplitudes of eating rhythms and amplitudes and phases of locomotor activity rhythms differed by diet and/or estradiol treatment (OriginLab Pro). When the interaction was significant, Tukey post-hoc analyses were conducted.

## Results and discussion

### Treatment with exogenous estradiol does not inhibit diet-induced obesity in male mice

**Fig 1:**
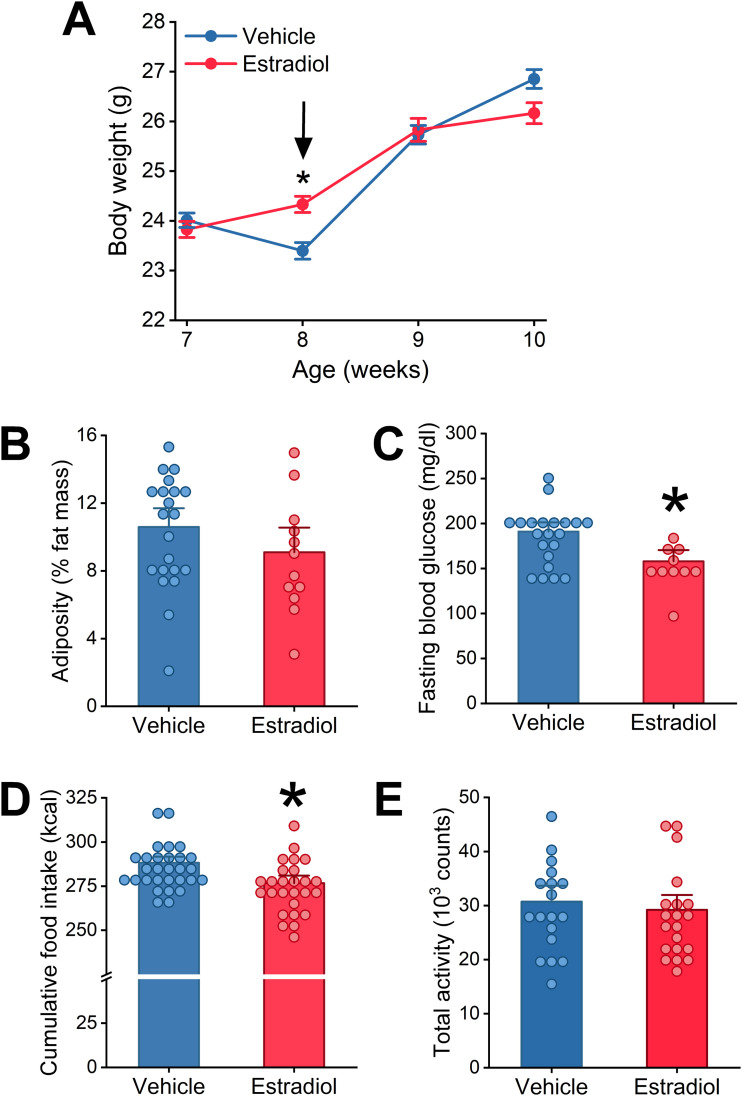
Treatment with exogenous estradiol does not inhibit body weight gain in male mice fed high-fat diet. At 7 weeks old, male C57BL/6J mice were implanted with Silastic tubing containing either vehicle (blue, n = 32) or 17β-estradiol (red, n = 26) and fed low-fat diet for 1 week and then high-fat diet for 2 weeks. Body weight (A, two-way RM ANOVA: Treatment *p* = 0.855, Age *p* < 0.001, Treatment*Age *p* < 0.001) was measured weekly. The arrow indicates the switch to high-fat diet. Adiposity (B, *t*-tes*t p* = 0.228) and fasting blood glucose (C, *t*-tes*t p* = 0.007) were measured at 10 weeks old. Cumulative food intake (D, *t*-tes*t p* = 0.002) and total activity counts (E, Mann-Whitney *p* = 0.500) were measured from 7-10 weeks old. *p < 0.01.

Female mice are resistant to diet-induced obesity in part because their circulating estrogens increase the amplitudes of their eating behavior rhythms [25,26,35,36]. To determine whether systemic estradiol regulates the eating behavior rhythm and diet-induced obesity in males, we implanted male C57BL/6J mice with Silastic tubing that contained either estradiol or vehicle. The dose of estradiol was approximately 10 times the level of estradiol in cycling females during proestrus. We chose this dose because a prior study found that chronic treatment of male mice with approximately this dose of estradiol inhibited high fat diet-induced obesity [37–39]. The male mice in this study were fed low-fat diet for 1 week and high-fat diet for 2 weeks (Fig 1). Males gained weight during the study (RM ANOVA, main effect of age *p* < 0.001), but estradiol treatment did not affect body weight (Fig 1A: RM ANOVA, main effect of treatment *p* = 0.855), adiposity (Fig 1B *t*-test *p* = 0.228), nor lean mass (S1 Fig) in males fed high-fat diet. Body weight differed at 8 weeks old before the mice were fed high-fat diet, which may be due to the effects of estradiol on recovery from surgery (RM ANOVA age*treatment *p* < 0.001, post-hoc *p* < 0.001 at 8 weeks; S1 Table). Fasting blood glucose was reduced by estradiol treatment in males (Fig 1C, *t*-test *p* = 0.007).

Since energy balance is regulated in part by energy intake and energy expenditure, we next determined whether estradiol treatment affected energy intake and activity in males. Male mice treated with estradiol consumed slightly fewer total calories than vehicle treated mice (Fig 1D, *t*-test *p* = 0.002, S1 Fig). Activity levels were not affected by estradiol treatment (Fig 1E, Mann-Whitney *p* = 0.500, S1 Fig). These data demonstrate that estradiol treatment has a small effect on energy intake in males.

Our results are consistent with a prior study that showed no short-term effect of estradiol treatment on body weight in male mice fed a high-fat diet [39]. In the previous study, chronic estradiol treatment and high-fat feeding for longer than 9 weeks were necessary for estradiol treatment to inhibit obesity [39]. There was no effect of estradiol treatment at timepoints before 9 weeks of high-fat feeding [39]. It is notable that both our study and the prior study of chronic estradiol treatment in males used estradiol doses that would be supraphysiological in females. In females, treatment with supraphysiological estradiol levels inhibited diet-induced obesity and increased leptin sensitivity [40–42]. Based on prior studies in males and females, we predicted that supraphysiological estradiol levels in males would inhibit diet-induced obesity and reveal effects of estradiol on circadian rhythms. However, it is possible that treating males with a dose of estradiol that is physiological in females (that mimics circulating estradiol levels during proestrus) would affect diet-induced obesity.

Overall, our data suggest that diet-induced obesity is regulated in part by distinct pathways in males and females, since our prior experiments using short-term estradiol treatment showed that estradiol treatment protected females from diet-induced obesity [25].

### Estradiol treatment of male mice does not affect eating behavior rhythms during high-fat diet feeding

**Fig 2:**
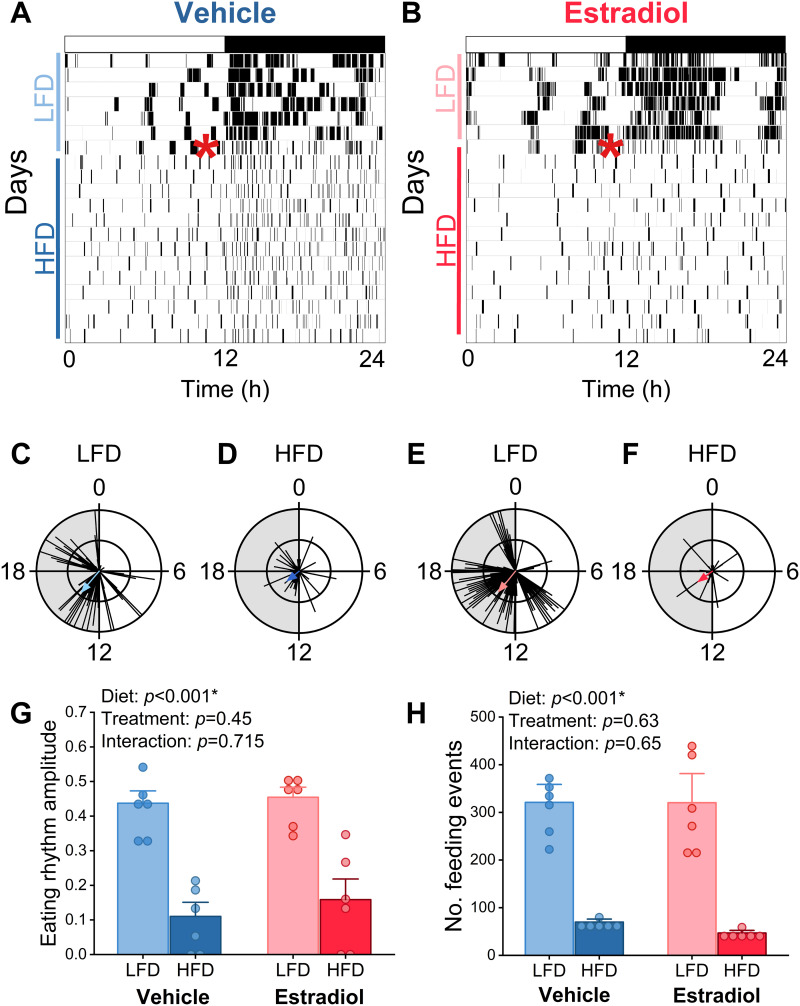
Estradiol does not affect eating behavior rhythms of male mice. Representative actograms (A,B, 6 minute bins; red asterisk indicates the switch from LFD to HFD) and circular histograms (C-F, 10 minute bins; scale: inner circle = 5, outer circle = 10) of eating behavior from male mice implanted with Silastic tubing containing either vehicle (A, C, D: blue, n = 6) or 17β-estradiol (B, E, F: red, n = 6). The eating rhythm amplitudes (G) were determined by averaging the last 3 days of LFD feeding (days 5-7) and the last 3 days of HFD feeding (days 19-21; two-way ANOVA: diet *p* < 0.001, treatment *p* = 0.45, diet*treatment *p* = 0.715). The number of daily feeding events (H) were average during the last 3 days of LFD (days 5-7) and HFD (19-21) feeding (two-way ANOVA, diet *p* < 0.0001, treatment *p* = 0.63, diet*treatment *p* = 0.65).

We previously found that circulating estradiol was necessary and sufficient for females to maintain high amplitude eating behavior rhythms during high-fat diet feeding [25,26]. In contrast, treatment with exogenous estradiol in male mice had no effect on the amplitudes of eating behavior rhythms (Fig 2, individual days shown in S2 and S3 Figs). Low-fat diet consumption was similarly consolidated during the night in males treated with vehicle and estradiol (Fig 2A, 2B, 2C, 2E). The amplitudes of eating behavior rhythms in males treated with vehicle and estradiol were both markedly reduced by high-fat feeding (Fig 2A, 2B, 2D, 2F, 2G, two-way ANOVA, main effect diet *p* < 0.001). These data demonstrate that eating behavior rhythms are not responsive to circulating estradiol in males, which contrasts the amplitude-enhancing effect of estradiol in females. We also measured the effect of estradiol treatment on the number of daily eating events, which is a marker of homeostatic responses to high-fat food intake. There was no effect of estradiol treatment on the total number of feeding events, since high-fat feeding reduced eating events in both groups (Fig 2H, two-way ANOVA, main effect diet p < 0.001).

### Estradiol treatment does not affect locomotor activity rhythms of male mice fed high-fat diet

**Fig 3:**
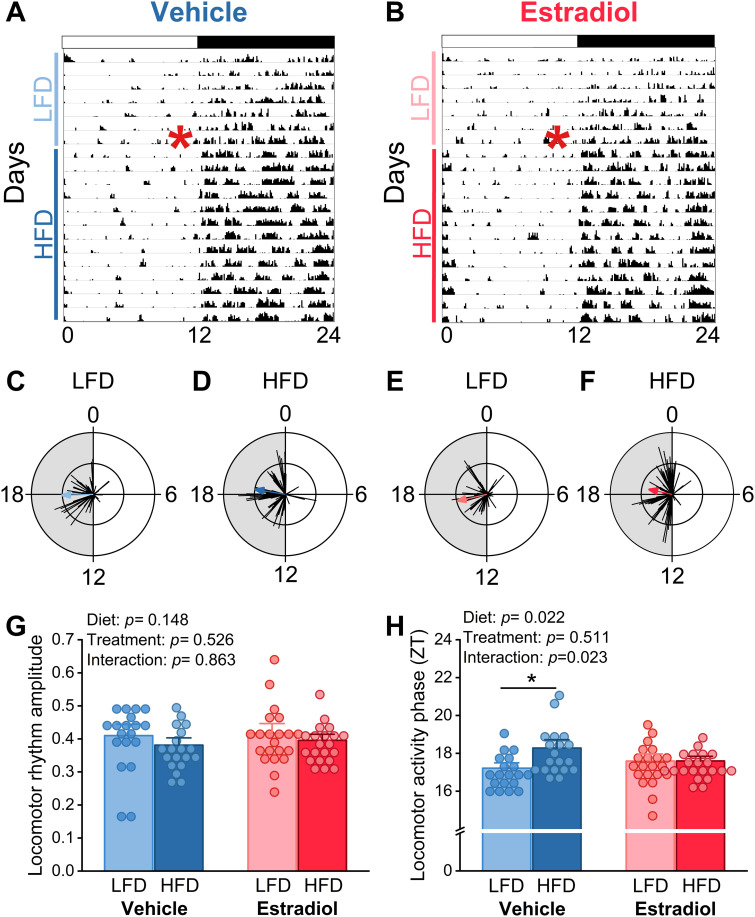
Estradiol does not affect locomotor activity behavior rhythms of male mice. Representative actograms (A,B, 6 minute bins, red asterisk represents the change from LFD to HFD) and circular histograms (C-F, scale: inner circle = 35, outer circle = 70) of locomotor activity from male mice implanted with Silastic tubing containing either vehicle (A, C, D: blue, n = 19) or 17β-estradiol (B,E, F: red, n = 21). Mean amplitudes (G, two-way ANOVA, diet *p* = 0.148, treatment *p =* 0.526, and diet*treatment *p =* 0.863) and phases (H, two-way ANOVA, diet *p* = 0.022, treatment *p* = 0.511, diet*treatment *p* = 0.023) of locomotor activity rhythms were analyzed.

The amplitudes of the activity rhythms in female mice with circulating estrogens increases when females are fed high-fat diet [25]. In contrast, the locomotor activity of intact males and ovariectomized females is decreased during high-fat diet feeding [26,31]. We found there were no differences between vehicle- (Fig 3, all mice shown in S4 and S5 Figs) and estradiol- (Fig 3, all mice shown in S6 and S7 Figs) treated males in the amplitudes (Fig 3G, two-way ANOVA: diet: *p =* 0.148, treatment: *p =* 0.526, and diet*treatment: *p* = 0.863) of their locomotor activity rhythms. The phases of the locomotor activity rhythms were delayed by high-fat diet feeding in vehicle-treated, but not estradiol-treated males (Fig 3H, two-way ANOVA: diet: *p =* 0.022, treatment: *p* = 0.511, and diet*treatment: *p* = 0.023, post-hoc test, *p* = 0.01). Activity offsets did not differ between estradiol and vehicle-treated male mice (S8 Fig, two-way ANOVA, diet: *p* = 0.07, treatment: *p* = 0.43, and diet*treatment: *p* = 0.49). Thus, estradiol treatment does not enhance activity rhythms during high-fat feeding in males as it does in females.

### Exogenous estradiol does not affect circadian organization in male mice

**Fig 4:**
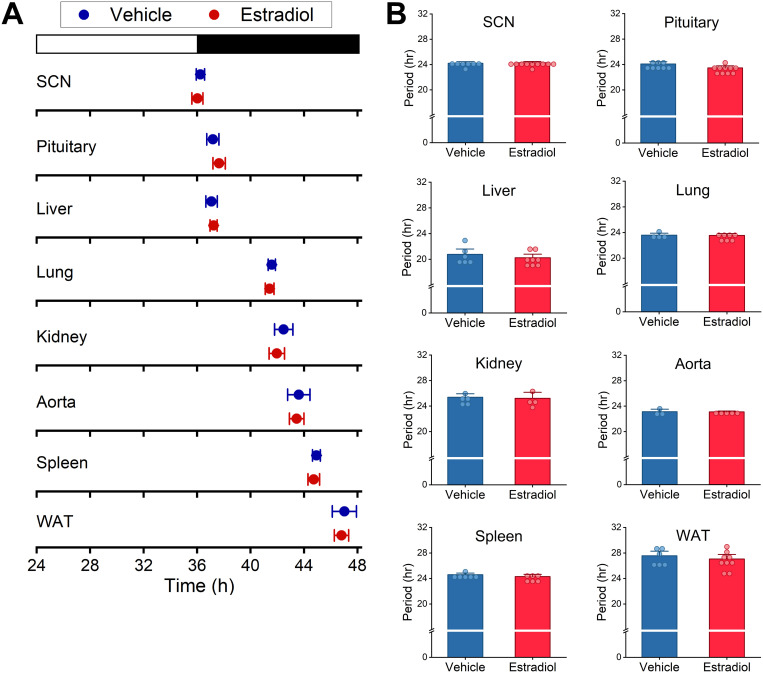
Exogenous estradiol treatment does not affect circadian organization in male mice fed high-fat diet. PER2::LUC males were implanted with Silastic tubing containing vehicle (blue) or estradiol (red) and fed high-fat diet for 2 weeks. Tissue explants were cultured and bioluminescence was measured. The mean phases of bioluminescence rhythm peaks were plotted relative to last lights on **(A)**. The white and black bars represent the times of light and dark, respectively. The periods of peripheral tissues were measured in estradiol and vehicle-treated males **(B)**. SCN: Suprachiasmatic Nucleus, WAT: White adipose tissue.

High-fat diet feeding advances the phase of the liver circadian clock in male mice but does not affect the phases of circadian clocks in the SCN or other peripheral tissues [29]. Therefore, high-fat feeding causes circadian misalignment by altering the phase of the liver circadian clock in males. There is a sex difference in the effect of high-fat feeding on the liver clock that is regulated by circulating estradiol in females. The phase of the liver circadian clock is not affected by high-fat diet feeding in gonadally-intact females and ovariectomized females treated with exogenous estradiol [25,26]. Thus, we investigated whether exogenous estradiol treatment affected the phases or periods of central and peripheral tissues in male mice fed high-fat diet (Fig 4A-4B and S2 Table). We found that the phases of circadian clocks in the SCN and peripheral tissues, including the liver, in male mice fed high-fat diet did not differ between mice treated with estradiol or vehicle (Fig 4A, S2 Table). Estradiol treatment did not affect the periods of PER2::LUC rhythms in males fed high-fat diet (Fig 4B). These data demonstrate that tissue circadian clocks in males and females do not respond similarly to estradiol treatment during high-fat diet feeding.

## Conclusions

The daily eating rhythm, or the time of day when food is consumed, has emerged as a factor that regulates obesity [25,36,43,44]. We previously showed that there is a sex difference in the effect of high-fat diet feeding on the daily eating rhythm. Eating rhythms in males were disrupted by high-fat diet feeding while eating rhythms in females were not [26,29,31]. Circulating estradiol was the regulator of the eating rhythm in female mice [25]. In this study, we performed a similar study in male mice and investigated the effects of circulating exogenous estradiol on eating rhythms in males fed high-fat diet. We found that estradiol, when administered in adulthood, did not regulate the eating rhythm in male mice during 2 weeks of high-fat diet feeding. Thus, unlike female mice, estradiol did not increase the amplitude of the daily eating rhythm. It is possible that longer duration treatment with estradiol and high-fat feeding may have revealed estradiol regulation of the eating rhythm in males. A prior study found that chronic estradiol treatment (9 weeks) inhibited high-fat diet-induced obesity in male mice [39]. In male mice, we found that diet-induced obesity is associated with disrupted eating rhythms [45]. Thus, it is possible that estradiol would regulate the daily eating rhythm during chronic high-fat feeding.

Taken together, these data demonstrate that exposure to estradiol during adulthood alone does not account for the sex difference in the daily rhythms underlying diet-induced obesity in mice. Moreover, our findings suggest that during development there is sexual differentiation of brain circuits that regulate eating rhythms. Organization of these brain circuits may rely on the unique hormonal milieus in male and females during the critical period of brain sexual differentiation [46].

## Supporting information

S1 Fig Metabolic parameters in males treated with estradiol compared to those treated with vehicle. Fat mass (A, *t*-test, *p* = 0.24) and lean mass (B, Mann-Whitney test, *p* = 0.45) in males treated with estradiol (red) compared to vehicle (blue). Weekly energy intake (C, two-way RM ANOVA, treatment *p* = 0.014, time *p* < 0.0001, treatment*time *p* = 0.027, Bonferroni post-hoc *p* = 0.005 at week 2) and weekly activity (D, two-way RM ANOVA, treatment *p* = 0.56, time *p* < 0.0001, treatment*time *p* = 0.21) in males treated with estradiol compared to vehicle. *p < 0.01.
(TIF)

S2 Fig Eating behavior rhythms are disrupted by high-fat feeding in vehicle-treated male mice. Circular histograms of eating behavior for 3 days of low-fat feeding (days 5–7; left: LFD) or 3 days of high-fat feeding (days 19–21; right: HFD) for each mouse (individual mice shown in A-F). The lengths of the vectors are the amplitudes of the eating behavior rhythms. When eating behavior was arrhythmic (Rayleigh test *p* > 0.05), then the amplitude was 0 and there was no vector shown on the circular histogram. Circular histogram scale: inner circle, 5 eating events; outer circle, 10 eating events. Lights were on from ZT0–12 (right side) and off from 12 to 24 (shaded left sides).
(TIF)

S3 Fig Eating behavior rhythms are disrupted by high-fat feeding in male mice treated with exogenous estradiol. Circular histograms of eating behavior for 3 days of low-fat feeding (days 5–7; left: LFD) or 3 days of high-fat feeding (days 19–21; right: HFD) for each mouse (individual mice shown in A-F). The lengths of the vectors are the amplitudes of the eating behavior rhythms. When eating behavior was arrhythmic (Rayleigh test *p* > 0.05), then the amplitude was 0 and there is no vector shown on the circular histogram. Circular histogram scale: inner circle, 5 eating events; outer circle, 10 eating events. Lights were on from ZT0–12 (right side) and off from 12 to 24 (shaded left sides).
(TIF)

S4 Fig General locomotor activity of male mice treated with vehicle. General locomotor activity was recorded continuously by passive infrared sensors for 7 days of low-fat diet feeding and then 14 days of high-fat diet feeding. Circular histograms and Rayleigh tests from day 7 (last day of LFD) and day 21(last day of HFD) were analyzed (see Fig. S5). Cumulative activity counts (shown in Fig. 1E) were measured for mice in B-S, but were not measured for the mouse shown in panel A because of one night of missing activity counts.
(TIF)

S5 Fig Amplitudes and phases of general locomotor activity rhythms in males treated with vehicle. Circular histograms for the last day of low-fat diet (LFD; day 7) and the last day of high fat diet (HFD; day 21) feeding for each mouse. The length and direction of the vector are the amplitude and phase of the locomotor activity rhythm, respectively. Circular histogram scale: inner circle, 35 activity counts; outer circle, 70 activity counts. Lights were on from ZT0–12 (right side) and off from 12 to 24 (shaded left sides). The mice in A-S correspond to the actograms shown in Fig. S3A-S.
(TIF)

S6 Fig General locomotor activity of male mice treated with estradiol. General locomotor activity recorded continuously by passive infrared sensors for 7 days of low-fat diet feeding and then 14 days of high-fat diet feeding. Circular histograms and Rayleigh tests from day 7 (last day of LFD) and day 21(last day of HFD) were analyzed (Fig. S6). Cumulative activity counts (shown in Fig. 1E) were measured for mice in A-Q and S-U, but were not for the mouse shown in R because of one night of missing activity counts.
(TIF)

S7 Fig Amplitudes and phases of general locomotor activity rhythms in males treated with estradiol. Circular histograms were made for the last day of low-fat diet (LFD; day 7) and last day of high fat diet (HFD; day 21) for each mouse. The length and direction of the vector are the amplitude and phase of the locomotor activity rhythm, respectively. Circular histogram scale: inner circle, 35 activity counts; outer circle, 70 activity counts. Lights were on from ZT0–12 (right side) and off from 12 to 24 (shaded left sides). The mice in A-U correspond to the actograms shown in Fig. S4A-U.
(TIF)

S8 Fig Activity offsets do not differ between males treated with estradiol compared to those treated with vehicle. Activity offsets (in ZT) were measured on the last day of low-fat feeding (day 7) and the last day of high-fat feeding (day 21; Two-way ANOVA, diet *p* = 0.07, treatment *p* = 0.43, and diet*treatment *p* = 0.49). Activity offsets were measured with ClockLab Analysis software.
(TIF)

S1 Table Weekly percent body weight changes (mean ± SEM). (DOCX)

S2 Table Phases of bioluminescence rhythms measured from *ex vivo* tissues. (DOCX)
