# Supplementary material for: Exogenous estradiol does not regulate daily metabolic rhythms underlying diet-induced obesity in male mice
Source: PLoS One. 2026 Mar 17;21(3):e0343513. doi: 10.1371/journal.pone.0343513 (PMC12994778; doi:10.1371/journal.pone.0343513)
Supplement: S1 Table — (DOCX) [file pone.0343513.s009.docx]

| **S1 Table. Weekly percent body weight changes (mean ± SEM)** | | | |
| --- | --- | --- | --- |
|  | Week 1 (LFD) | Week 2 (HFD) | Week 3 (HFD) |
| Vehicle | -2.6 ± 0.54 | 10.02 ± 0.63 | 4.27 ± 0.50 |
| Estradiol | 2.15 ± 0.54 | 6.18 ± 0.74 | 1.34 ± 0.48 |
