# Supplementary material for: Exogenous estradiol does not regulate daily metabolic rhythms underlying diet-induced obesity in male mice
Source: PLoS One. 2026 Mar 17;21(3):e0343513. doi: 10.1371/journal.pone.0343513 (PMC12994778; doi:10.1371/journal.pone.0343513)
Supplement: S2 Table — (DOCX) [file pone.0343513.s010.docx]

**S2 Table. Phases of bioluminescence rhythms measured from *ex vivo* tissues.**

**Vehicle Estradiol**

**Tissue (mean ± SEM) (n) (mean ± SEM) (n) Statistics (*t*-test)**

Phase SCN 36.42 ± 0.31 (7) 36.02 ± 0.36 (10) *t* = 0.80, *p* = 0.43

Pituitary 37.12 ± 0.53 (8) 37.46 ± 0.43 (10) *t* = -0.51, *p* = 0.62 Liver 36.03 ± 0.31 (7) 35.88 ± 0.29 (8) *t* = 0.36, *p* = 0.73

Lung 41.44 ± 0.18 (7) 41.55 ± 0.37 (9) *t* = 0.64, *p* = 0.53

Kidney 42.03 ± 0.28 (6) 41.92 ± 0.21 (8) *t* = 0.32, *p* = 0.75

Aorta 43.53 ± 0.67 (6) 43.94 ± 0.38 (8) *t* = -0.58, *p* = 0.57

Spleen 44.93 ± 0.22 (7) 44.69 ± 0.40 (10) *t* = 0.48, *p* = 0.64

WAT 48.22 ± 0.70 (7) 47.33 ± 0.80 (9) *t* = 0.81, *p* = 0.43
